# Complexities due to single-stranded RNA during antibody detection of genomic rna:dna hybrids

**DOI:** 10.1186/s13104-015-1092-1

**Published:** 2015-04-08

**Authors:** Zheng Z Zhang, Nicholas R Pannunzio, Chih-Lin Hsieh, Kefei Yu, Michael R Lieber

**Affiliations:** USC Norris Comprehensive Cancer Ctr. Molecular and Computational Biology Program, Department of Biological Sciences, University of Southern California Keck School of Medicine, 1441 Eastlake Ave., Rm. 5428, Los Angeles, CA 90089-9176 USA; Departments of Pathology, Biochemistry & Molecular Biology; Molecular Microbiology & Immunology; Urology, University of Southern California Keck School of Medicine, 1441 Eastlake Ave., Rm. 5428, Los Angeles, CA 90089-9176 USA; Department of Microbiology and Molecular Genetics, Michigan State University, 5175 Biomedical Physical Sciences, East Lansing, MI 48824 USA

**Keywords:** Immunogloblulin heavy chain class switch recombination, R-loop, Immunoprecipitation, S9.6 monoclonal antibody, Nucleic acid structure

## Abstract

**Background:**

Long genomic R-loops in eukaryotes were first described at the immunoglobulin heavy chain locus switch regions using bisulfite sequencing and functional studies. A mouse monoclonal antibody called S9.6 has been used for immunoprecipitation (IP) to identify R-loops, based on the assumption that it is specific for RNA:DNA over other nucleic acid duplexes. However, recent work has demonstrated that a variable domain of S9.6 binds AU-rich RNA:RNA duplexes with a K_D_ that is only 5.6-fold weaker than for RNA:DNA duplexes. Most IP protocols do not pre-clear the genomic nucleic acid with RNase A to remove free RNA. Fold back of ssRNA can readily generate RNA:RNA duplexes that may bind the S9.6 antibody, and adventitious binding of RNA may also create short RNA:DNA regions. Here we investigate whether RNase A is needed to obtain reliable IP with S9.6.

**Findings:**

As our test locus, we chose the most well-documented site for kilobase-long mammalian genomic R-loops, the immunoglobulin heavy chain locus (IgH) class switch regions. The R-loops at this locus can be induced by using cytokines to stimulate transcription from germline transcript promoters. We tested IP using S9.6 with and without various RNase treatments. The RNase treatments included RNase H to destroy the RNA in an RNA:DNA duplex and RNase A to destroy single-stranded (ss) RNA to prevent it from binding S9.6 directly (as duplex RNA) and to prevent the ssRNA from annealing to the genome, resulting in adventitious RNA:DNA hybrids. We find that optimal detection of RNA:DNA duplexes requires removal of ssRNA using RNase A. Without RNase A treatment, known regions of R-loop formation containing RNA:DNA duplexes can not be reliably detected. With RNase A treatment, a signal can be detected over background, but only within a limited 2 or 3-fold range, even with a stable kilobase-long genomic R-loop.

**Conclusion:**

Any use of the S9.6 antibody must be preceded by RNase A treatment to remove free ssRNA that may compete for the S9.6 binding by forming RNA:RNA regions or short, transient RNA:DNA duplexes. Caution should be used when interpreting S9.6 data, and confirmation by independent structural and functional methods is essential.

**Electronic supplementary material:**

The online version of this article (doi:10.1186/s13104-015-1092-1) contains supplementary material, which is available to authorized users.

## Findings

### Research hypothesis

Physical presence of long genomic R-loops in eukaryotes was first described at the IgH switch regions [1]. This genomic R-loop analysis was based on (a) bisulfite sequencing [1,2] and (b) oligonucleotide hybridization (colony lift hybridization) [3,4], and both were done with and without an RNase H challenge treatment to confirm the RNA:DNA conformation. Moreover, we did not observe any large effects of parallel RNase A treatment *in vivo* [1,3-5] or *in vitro* [6-8]. Our original description of kilobase long mammalian genomic R-loops was further built upon and had the advantage of several lines of independent evidence including (a) the large body of IgH switch DNA sequence and recombination junctional sequence information [9,10]; (b) many functional studies of IgH switch region transcription [11]; (c) concurrent studies of IgH switch region orientation [12,13]; and (d) detailed *in vitro* biochemical studies of transcription through switch regions [6-8,14-17]. Therefore, these well-documented regions with R-loops are ideal positive control targets of IP using S9.6 antibody.

While S9.6 antibody can recognize RNA:DNA duplexes [18-28], complete characterization of the binding specificity of S9.6 was initially limited to ELISA measurements on its binding to long nucleic acid duplexes [29]. Such ELISA measurements can be complicated by multiple antibodies binding to a single long duplex. This multi-antibody complex would reflect the combination of affinities of multiple antibodies [(K_D_)^n^, where n = the number of antibodies bound to a given duplex]. Recent work has shown that a single-chain variable domain of the S9.6 antibody can bind RNA:RNA duplexes with an affinity that is only 5.6-fold weaker than to RNA:DNA duplexes, raising the serious concern that S9.6 can indeed cross react with RNA species [30]. Most of the studies that have used S9.6 to identify R-loops in eukaryotes have not used RNase A to eliminate any artifacts due to free RNA [18-28,30,31], which might be present during cell lysis and/or harvest of the nucleic acid. In addition, free RNA can reanneal with the template DNA during transient breathing of the DNA, and short RNA:DNA hybrids also exist during DNA replication at RNA primer annealing sites. Here we examine the complexities of R-loop analysis when using the S9.6 antibody with and without various RNase treatments. We used the mouse B-cell line, CH12F3.2a [32], which is able to specifically and efficiently switch to IgA upon cytokine stimulation, thus providing the only widely-accepted extended R-loop as a positive control [33-35].

### Methods

#### Cell culture

CH12F3.2a and its derivative cells were cultured in RPMI medium supplemented with 10 % FCS and 50 μM β-mercaptoethanol [36]. For experiments specifying cytokine stimulation, two million healthy CH12F3.2a cells at a density around 1×10^6^ cells/ml were supplemented with anti-CD40 (eBioscience Cat. No. 16-0404-86), IL-4 (R&D Cat. No. 404-ML-010) and TGF- β1 (R&D Cat. No. 240-B-002) for 24 hours.

#### S9.6 Purification

ATCC HB-8730 hybridoma line (generously provided by Bradley Cairns) was cultured in a CELLine 1000 bioreactor (Satorius Biotech, NY) according to manufacturer’s instructions. Harvested antibody (culture supernatant) was purified on a column packed with Protein G Sepharose 4 Fast Flow (GE Healthcare) equilibrated with 1x phosphate buffered saline (PBS).

#### S9.6 Immunoprecipitation and qPCR

Genomic DNA from CH12F3.2a cells with and without cytokine stimulation was prepared by overnight proteinase K digestion, phenol-chloroform extraction and ethanol precipitation. Genomic DNA was digested with EcoRI; importantly, RNase A was added at this step to prevent S9.6 antibody binding to RNA species in subsequent steps in the experiments with RNase A treatment (Phillips et al. [30]). Five microgram of digested genomic DNA were incubated with 5 μg of S9.6 antibody in 400 μl IP buffer (10 mM sodium phosphate pH 7.0, 140 mM NaCl, 0.1% Tween 20) for 2 h at 4°C with rotation. Ten microliter pre-blocked Dynabeads (Invitrogen 10004D) were added to the mixture and gently rotated at 4°C for 2 h. The beads were washed with IP buffer three times before the bead-bound DNA was recovered by proteinase K treatment overnight, phenol-chloroform extraction, and ethanol precipitation. The recovered DNA was then analyzed by quantitative real-time PCR (qPCR) using TaqMan probes. The sequence details of the primers and probes are shown in Additional file 1: Table S1. For the beta-actin gene, the probe for real-time PCR was: ZZ218 FAM-CACCGCAAGTGCTTCTAGGCGGAC-BHQ, and the primers were: ZZ221 - GAGTCCGGCCCCTCCAT and ZZ222 - GGTTTTGTCAAAGAAAGGGTGTAAA. Each IP experiment was done in triplicate, and each qPCR is done in duplicate.

### Results

#### R-loops at the Mammalian IgH Switch Regions

**Figure 1:**
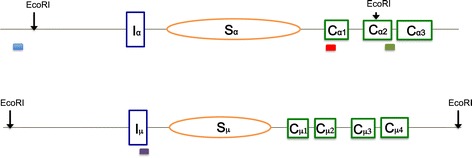
**Mammalian IgH switch regions.** Genomic configuration around Sα (top panel) and Sμ (bottom panel) is as illustrated. Orange ovals represent switch regions, and green rectangles represent constant regions. Restriction enzyme (EcoRI) sites are shown on top. Solid red, blue, green, and purple bars are the locations of qPCR amplicons in the DNA regions containing Sα, upstream of Sα, downstream of Sα and Sμ, respectively.

We stimulated CH12F3.2a cells (which are capable of robust cytokine-dependent CSR in culture) for 24 hr before genomic DNA was harvested and digested in preparation for immunoprecipitation (IP) as described previously [34]. The transcription level of the IgH Sα in CH12F3.2a cells increased slightly upon cytokine stimulation [37]. Four different DNA regions were chosen for qPCR analysis after IP. The first two are the IgH Sα region and IgH Sμ region, which we have shown to form R-loops within cells, based on genomic bisulfite sequencing [3,33,38]. IgH Sμ is already highly transcribed without cytokine stimulation; therefore, cytokine stimulation does not increase R-loop formation in this region of the IgH locus. We chose two nearby regions as controls. The first control region is located upstream of the adjacent transcriptional promoters. The second control region is located far downstream (Figure 1; note IgH locus with EcoRI sites and qPCR amplicons). These controls reside within different restriction fragments that are distinct from the Ig class switch regions.

**Figure 2:**
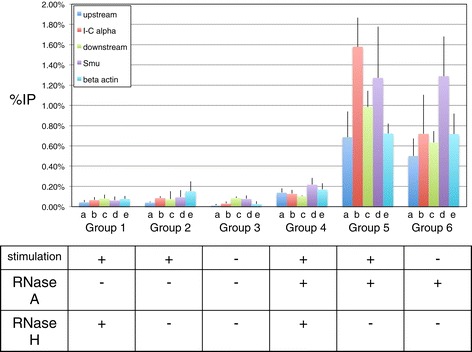
**S9.6 IP at Mammalian IgH switch regions requires RNase a pre-treatment of the harvested genomic nucleic acid.** Immunoprecipitation with the S9.6 antibody was performed on stimulated or unstimulated wild type CH12F3.2a cells. The genomic DNA samples were pre-treated with RNase A before IP for Groups 4 through 6. Cytokine stimulation and RNase treatments are specified at the bottom. Half of the genomic DNA was pretreated with RNase H before IP. Background signals from mock samples with no antibody were subtracted. Values were normalized to the total input DNA to calculate the pull-down percentage. Three independent IP experiments were performed for each condition. Error bars represent standard error of the mean (SEM). The Sα region in Group 5 shows the highest level of S9.6 IP, though this level is only of borderline significance (p = 0.08) compared to the region at the same locus, but upstream of the EcoRI site. This reflects the limited signal to noise detection capability of S9.6.

In these IP experiments [34], we find that the pull down of the DNA fragment containing the Sα region is more than two fold higher with cytokine stimulation (Figure 2, Group 5 versus Group 6, column b) while other regions showed no or very little change (Figure 2, Group 5 versus Group 6, columns a, c, and d). The downstream region, which showed a very small increase of pull down in the stimulated cells, may be due to the R-loop formation in the IgH Sα affecting the efficient digestion of EcoRI site in the DNA fragment. To further confirm that the signals that we detected arose from RNA:DNA hybrids, we treated the DNA samples with RNase H before the IP experiment, an endonuclease that specifically degrades RNA in RNA:DNA duplexes. Upon RNase H treatment, the signals from all regions that we examined decreased 5 to 10-fold (Figure 2, compare Group 5 versus Group 4) indicating that the IP signals are indeed due to RNA:DNA hybrids.

It is surprising that we detected signals clearly above the background within the fragments far upstream and downstream of the alpha locus even though these signals do not change with cytokine stimulation. We have previously documented that the region upstream of the germline transcript promoter for the IgH alpha locus and the downstream region do not harbor any R-loops, regardless of cytokine treatment [1,3,4,33]. We confirmed that this was not merely due to incomplete restriction digestion because the signal is present after multiple rounds of digestion.

We also examined the beta-actin gene in the DNA samples from the same IP experiments. The beta-actin gene is at another location in the genome, and it has not been documented to have any extended R-loop by physical methods. The signal at the beta-actin gene is similar to all regions other than IgH Sα (Figure 2, all Groups, column e), and this signal also does not change with cytokine stimulation. These findings indicate that while the S9.6 antibody binds to well-documented R-loops in the IgH Sα, this antibody may also bind to DNA regions that are clearly known to have no extended R-loops. In addition to binding to bona fide extended R-loops [1,3,4,33], it is possible that the S9.6 antibody can bind to very small RNA:DNA hybrids, such as those formed by RNA primers for DNA replication and the RNA transcripts that may transiently reanneal back to the DNA template during or before genomic DNA is harvested. This may account for the RNase H-sensitivity of these IP signals at locations outside of IgH switch regions (Figure 2, Group 4).

Taken together, the S9.6 antibody appears to have good specificity for binding to RNA:DNA hybrids, but its ability to distinguish extended R-loops versus small RNA:DNA hybrids arising during DNA replication (RNA primer sites) or from other causes is highly uncertain. With the potential high background due to the antibody binding to non-R-loop RNA:DNA hybrids, the dynamic range of the R-loop signal is greatly affected.

#### Cellular RNA complicates R-loop detection

In all the IP experiments of DNA samples without RNase A treatment regardless of cytokine stimulation, qPCR did not detect any signal above background in any regions examined (Figure 2, Groups 1 to 3). This is also surprising since RNase A does not affect RNA:DNA hybrids. This finding indicates that the vast amount of RNA in the DNA samples can indeed affect S9.6 binding to RNA:DNA hybrids. While the mechanism involved in this interference is undetermined in our experiments, the increase of signals in experiments with RNase A treatment reflects the possibility that RNA species in the DNA may titrate out the S9.6 antibody (Figure 2, Groups 2 and 3 versus Groups 5 and 6). It is possible that the free RNA can fold back on itself to form RNA:RNA duplexes, which is known to compete with RNA:DNA for S9.6 binding [30]. This finding strongly indicates that RNase A pre-treatment is necessary for the S9.6 antibody to clearly detect the presence of RNA:DNA duplexes.

### Conclusions

The IgH class switch region R-loops can be up to kilobases in length, and thus provide the largest possible target upon which to test the S9.6 antibody for detection of RNA:DNA duplex in the mammalian genome [1,2]. Despite such a large target, we found that the detection of these kilobase R-loops is less than 3-fold above background with borderline significance even with the essential pre-treatment of genomic nucleic acid with RNase A. We also found that the regions known to have no extended R-loops can also be pulled down by the S9.6 antibody. We suspect that abundant free RNA transcripts can reanneal to the template DNA strands to form RNA:DNA hybrids for short lengths at low levels throughout the genome. Also, RNA primers generated during the course of physiologic cellular DNA replication in the sub-population of proliferating cells in S phase might also have short (~20 bp) RNA:DNA duplexes that become sites of S9.6 binding. It is likely that these two forms of RNA:DNA hybrids compete with the long and stable R-loops, such as at the IgH switch locus, and result in promiscuous S9.6 binding on a genome-wide basis in IP experiments (Additional file 1: Figure S1) These findings suggest that while the S9.6 antibody binds to RNA:DNA hybrids with good specificity, its ability to distinguish extensive R-loops of biological significance and very short RNA:DNA hybrids due to transient RNA reannealing to DNA is uncertain. This not surprising since the S9.6 antibody was generated using short RNA:DNA hybrids. We also found that RNA remaining in the genomic DNA harvested from the cells affects S9.6 binding to RNA:DNA hybrids dramatically. It is quite common that free RNA folds back on itself to form RNA:RNA duplexes, which is known to compete with RNA:DNA for S9.6 binding [30]. This binding competition would potentially contribute to the low level of the IP signal when RNase A is not used and thus RNA:RNA duplexes are abundant. Removal of cellular RNA with RNase A reduces this competition of the antibody, thereby allowing S9.6 to bind more specifically to RNA:DNA hybrids. Based on our experiments, it is clear that identification of biologically important R-loops based solely on S9.6 is insufficient, especially when RNase A is not used prior to the assay. Findings in such studies should always be accompanied by chemical probing [1,2], quantitation of the number of alleles involved in the R-loop formation [3,4], and functional studies showing biological effects [12,34,35]. Without rigorous confirmation using independent methods, conclusions drawn from such studies would be highly questionable.

#### Availability of supporting data

We will immediately provide anyone with our primary data.

## Additional file

Additional file 1: Figure S1. Structure and Origins of Targets of the S9.6 Antibody. In addition to the stable R-loops at mammalian IgH switch regions (A), short R-loop-like structure may also form when RNA transcripts anneal back to the DNA template strand during DNA breathing (B), or when primases synthesize RNA primers during DNA replication (C). RNA with secondary structure may also be recognized by S9.6 (D). **Table S1.** Real-Time PCR Oligonucleotides.

## Abbreviations

Ig: Immunoglobulin
CSR: Class switch recombination
IP: Immunoprecipitation
qPCR: Quantitative PCR (also termed real-time PCR)
