# Supplementary material for: Complexities due to single-stranded RNA during antibody detection of genomic rna:dna hybrids
Source: BMC Res Notes. 2015 Apr 8;8:127. doi: 10.1186/s13104-015-1092-1 (PMC4393563; doi:10.1186/s13104-015-1092-1)
Supplement: Additional file 1: Figure S1. — Structure and Origins of Targets of the S9.6 Antibody. In addition to the stable R-loops at mammalian IgH switch regions (A), short R-loop-like structure may also form when RNA transcripts anneal back to the DNA template strand during DNA breathing (B), or when primases synthesize RNA primers during DNA replication (C). RNA with secondary structure may also be recognized by S9.6 (D). Table S1. Real-Time PCR Oligonucleotides. [file 13104_2015_1092_MOESM1_ESM.pdf]

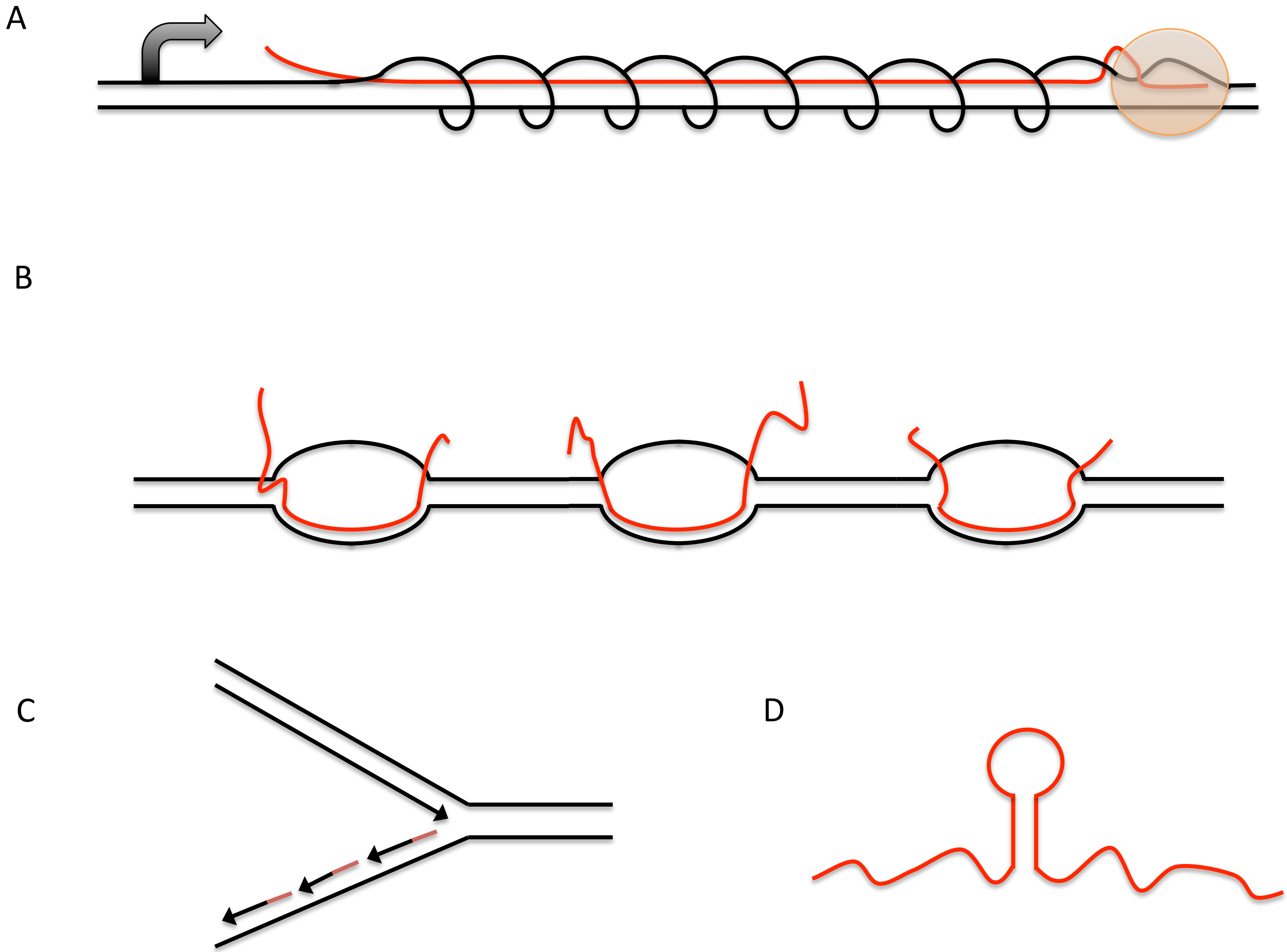

Suppl. Figure 1

Supplementary Table 1. Real-Time PCR Oligonucleotides.

	Sequence	Function
S9.6 ChIP		
ZZ304	[6-FAM]CCCAGTGATAATCGGCTGCCTGATTC[BHQ1a-6FAM]	probe for the Sα region
ZZ305	CCCACGAGCTCTGTCAAGTG	forward primer for the Sα region
ZZ306	GCCGGAAGGGAAGTAATCG	reverse primer for the Sα region
ZZ391	[6-FAM]CTGGCCACCTGGGCTCATCCC[BHQ1a-6FAM]	probe for the upstream fragment of the Sα region
ZZ389	CTCACCGATTTGACCTACCAGAT	forward primer for the upstream fragment of the Sα region
ZZ390	AGTGGTCCTGATGCCCATAGA	reverse primer for the upstream fragment of the Sα region
ZZ394	[6-FAM]CACAATTGCCAAAATCACAGGTGAGCC[BHQ1a-6FAM]	probe for the downstream fragment of the Sα region
ZZ392	CATCCTGAGTCTGACACCTTAACTG	forward primer for the downstream fragment of the Sα region
ZZ393	ACAATGTCCCGGGTATGCAT	reverse primer for the downstream fragment of the Sα region
RLB96	[6-FAM]AACTCTCCAGCCACAGTAATGACCCAGACA[BHQ1a-6FAM]	probe for the μ region
RLB 107	GCCACAGCTGTACAGAATTGAGA	forward primer for the μ region
RLB95	CAACCTTGTTCCCTTAATTTTGCT	reverse primer for the μ region
GLT Quantification		
ZZ274	CCTATGAAGGACACTCAACAACATTG	forward for the α region
ZZ275	CCGATTATCACTGGGTCACTTG	reverse primer for the α region
ZZ276	[6-FAM]ATCTACCCACTGACACTCCCACGAGCTC[BHQ1a-6FAM]	probe for the α region
ZZ277	TGCTCCCCGGGCTGTATT	forward primer for β-actin region
ZZ278	ACATAGGAGTCCTTCTGACCCATT	reverse primer for β-actin region
ZZ279	[6-FAM]ATCGTGGGCCGCCCTAGGCAC[BHQ1a-6FAM]	probe for the β-actin region
